# £25 and a biscuit: Women’s Health Research and Public Engagement in the UK

**DOI:** 10.1186/s40900-023-00519-1

**Published:** 2023-12-15

**Authors:** Alison Gabrielle Perry, Edward Mullins

**Affiliations:** 1 Imperial College London, London, UK; 2 The George Institute for Global Health, London, UK

## Abstract

It is over a  year since the Department of Health launched the Women’s Health Strategy for England and included the rally cry of “women’s voices”. However, methods and modes of the inclusion of women in their own health and health research still fall short. Patient and public engagement and involvement (PPIE) in women’s health research is considered a hallmark of a moral, ethical, and democratic society. Despite the call for the inclusion of “women’s voices” and “women’s stories”, approaches to PPIE often remain tokenistic and don’t address issues of representation, equality, and diversity or respond to wider racial inequalities in health. This past August marked the 103rd birthday of the late Henrietta Lacks who died of cervical cancer. Clones of her cells (HeLa cells) obtained without consent, continue to be used in laboratories around the world and serves as an ongoing reminder of dynamics and power in health research relationships with the public today. Historically, women have been mistreated and excluded from research and the reality that Black women in the UK remain 3.7 times more likely to die in childbirth makes the effectiveness of our research pathways critical (MBRRACE-UK, https://www.npeu.ox.ac.uk/mbrrace-uk). PPIE holds much potential to contribute to the improvement of shortcomings in maternity and women’s health, but not without deeper understanding of the ways in which engagement intrinsically, works. This article raises criticism of the current quality of engagement in women’s health research and calls for a redesign of our frameworks and the need to explore new configurations of the relationship between women’s health, research, and people.

## Abstract (plain language summary)

It is one year since the Department of Health launched the Women’s Health Strategy for England and included the rally cry of “women’s voices”. However, methods and modes of the inclusion of women in their own health and health research still fall short. Patient and public engagement and involvement (PPIE) in women’s health research is considered a hallmark of a moral, ethical, and democratic society. Despite the call for the inclusion of “women’s voices” and “women’s stories”, approaches to PPIE often remain tokenistic and don’t address issues of representation, equality, and diversity, or respond wider racial inequalities in health. This past August marked the 103rd birthday of the late Henrietta Lacks who died of cervical cancer. Clones of her cells (HeLa cells) obtained without consent, continue to be used in laboratories around the world and serves as an ongoing reminder of dynamics and power in health research relationships with the public today. Historically, women have been mistreated and excluded from research and the reality that Black women in the UK remain 3.7 times more likely to die in childbirth makes the effectiveness of our research pathways critical [9]. PPIE holds much potential to contribute to the improvement of shortcomings in maternity and women’s health, but not without deeper understanding of the ways in which engagement intrinsically, works. This article raises criticism of the current quality of engagement in women’s health research and calls for a redesign of our frameworks and the need to explore new configurations of the relationship between women’s health, research, and people.

## Commentary

### £25 and a biscuit

#### Women’s Health Research and Public Engagement in the UK

There is nothing intrinsically wrong with £25 and a biscuit. Many a motivational treat for contributing one’s time and opinion comes in less of an attractive package. Biscuits, however, vary enormously and not everyone likes a *Jammie Dodger*, so to speak*.* Suffice to say, that in the arena of women’s health research engagement, the layers of the proverbial *Bourbon biscuit* need pulling apart.

In health research, the moral impetus to include people in the development, design, and dissemination of research is not new. The origins of patient and public involvement and engagement in the United Kingdom can be found between the 1950s and 1970s [8, 12], and [15]. Since then, campaigns by patient groups including those harmed by research have continued to drive the inclusion of people forward. High-profile stories such as the Alder Hey Hospital scandal, involving the collection of deceased infant and child body parts over a 10-year period without parental consent or the thalidomide disaster of the 1960s have further sharpened issues and amplified public voice. More recently, the vaginal ‘mesh’ scandal exposed by the findings of the *Independent Medicines and Medical Devices Safety Review* (2020) remind us that this is living history [2]. Ultimately, the story of cervical cells obtained without consent from a 31-year-old African American woman dying of cancer in 1951, named Henrietta Lacks, provides the inarguable and immortal imperative for a relationship of *partnership* between the public and actors of health research.

Patient and public involvement in health research is now firmly considered a hallmark of an ethical, democratic, and moral society and the legitimacy of public engagement has stepped up to look the traditional authority of medical research in the eye [15]. Commitment to the inclusion of the public in healthcare is now enshrined in the Health and Social Care Act 2001 and the NHS Constitution. Both pieces of legislation, in principle, mandate the involvement of patients and the public in commissioning processes and decision-making.

In health research, patient and public involvement and engagement, known as PPIE, has become de rigueur for researchers, particularly when applying for funding. The National Institute of Health Research set the pace with the requirement to demonstrate and describe the nature of engagement with people for whom the research stands to benefit, at all stages of the research and UK standards for public involvement continue to evolve [11]. The Department of Health’s launch of the *Women’s Health Strategy for England* last summer included and made the call for ongoing inclusion of the “voices of women” [1]. Despite the nobility of the public engagement movement, however, “£25 and a biscuit” chimes as a euphemism for a persistent tokenism in women’s health research engagement, at risk of missing the point.

Critics of PPIE highlight the transactional and formulaic nature of encounters between researcher and ‘the public’ that can be rushed and chronically without follow-up [6]. Too frequently, PPIE encounters are set up at the behest of the researcher, on the researcher’s terms, turf, and time scales. The beck and call of engagement in research largely works in one direction. In the early setup phase of a women’s health community group in West London hoping to address some of these limitations, one woman likened the dynamic of research engagement and participation to an excavation in which people’s stories and experiences are *mined.* She declined to take part. The imagery of an open pit with the gemstones of research never being returned to the community from where they came may provide a useful analogy. Although women are increasingly invited and, in some cases, accepting to go to the ‘tea party’ of research, “*women’s voices*”, “*women’s lived experience”,* and “*women’s stories”* are not for sale. The economy of community engagement remains far from a social economic model of reciprocity and power-sharing, and some consider whether PPIE done poorly could do more harm than good [5].

Antithetically, the case has been made for public engagement, yet, largely speaking, we haven’t required of public engagement to prove its worth, before embracing it. Perhaps this speaks to the inherently *human* quality of engagement, however, the result is that existing theories and frameworks for understanding and constructing public engagement can be varied, absent, or confusing [7]. The moral and ethical feelgood factor of engagement in health research has created the situation where PPIE is widely called for, yet there remains a paucity of research which makes PPIE fully understood.

In obeyance of meeting requirements for what *looks* right, we’ve omitted to engage with the fundamental anatomy of engagement. Unanswered questions include: *how does engagement work, what works for whom, in what way and in what context? What is the nature of the relationship between engagement and women’s health? Are there better and different ways to do engagement? What does ‘success’ look like in PPIE?* If we profess to want to deal in the detail of people’s lives through research engagement, we need to know what we’re doing, why we’re doing, and how it works. If, in cultivating new theory, understanding, and creativity in women’s health engagement we need, also, to reach back to remember what it is to be human, then so be it.

For those of us with a research lens, this presents great opportunity. Proponents of the arts and health movement would argue that meaningful transformation in clinical research engagement including deeper listening will no doubt be led by greater creativity [3, 4, 14]. The vast potential of arts-based methods such as storytelling, film, and conversations that build trust is increasingly well-known, but we have barely scratched the surface [3, 13]. Greater representation of women’s experiences and inclusion of women’s voices could, indeed be harnessed by the arts and the arts may very well retrain our listening ear. It’s time, one could say, to move beyond the biscuit.

In August, the NIHR launched the second round of funding of the call for “*Developing Innovative, Inclusive, and Diverse Public Partnerships*”, which holds the potential for doing engagement differently [10]. This is a cue to move beyond current models of public engagement, to ones which include a seat for engagement at a different table, where the menu gets written. It’s time for the “£25 and a biscuit” model of public engagement to be remembered as a *Nice* stepping-stone we once took before a large leap to the redesign and reconfiguration of relationships in health, research, and people. In such a new space, we hope to find, rather than ticked boxes for the few, greater hope and health for, the many.

## Data Availability

Not applicable.
